# A Live Video Dyadic Resiliency Intervention to Prevent Chronic Emotional Distress Early After Dementia Diagnoses: Protocol for a Dyadic Mixed Methods Study

**DOI:** 10.2196/45532

**Published:** 2023-09-20

**Authors:** Sarah Bannon, Julie Brewer, Nina Ahmad, Talea Cornelius, Jonathan Jackson, Robert A Parker, Kristen Dams-O'Connor, Bradford C Dickerson, Christine Ritchie, Ana-Maria Vranceanu

**Affiliations:** 1 Brain Injury Research Center Department of Rehabilitation and Human Performance Icahn School of Medicine at Mount Sinai New York, NY United States; 2 Center for Health Outcomes and Interdisciplinary Research Department of Psychiatry Massachusetts General Hospital and Harvard Medical School Boston, MA United States; 3 Department of Medicine Columbia University Irvine Medical Center New York, NY United States; 4 Community Access, Recruitment, and Engagement Research Center Division of Clinical Research Massachusetts General Hospital and Harvard Medical School Boston, MA United States; 5 Biostatistics Center Department of Medicine Massachusetts General Hospital and Harvard Medical School Boston, MA United States; 6 Brain Injury Research Center Departments of Rehabilitation and Human Performance and Neurology Icahn School of Medicine at Mount Sinai New York, NY United States; 7 Frontotemporal Disorders Unit Departments of Neurology and Psychiatry Massachusetts General Hospital and Harvard Medical School Boston, MA United States; 8 Mongan Institute Center for Aging and Serious Illness and the Division of Palliative Care and Geriatric Medicine Department of Medicine Massachusetts General Hospital and Harvard Medical School Boston, MA United States

**Keywords:** dyad, dementia, emotional distress, intervention, diagnosis, telehealth

## Abstract

**Background:**

By 2030, approximately 75 million adults will be living with Alzheimer disease and related dementias (ADRDs). ADRDs produce cognitive, emotional, and behavioral changes for persons living with dementia that undermine independence and produce considerable stressors for persons living with dementia and their spousal care-partners—together called a “dyad.” Clinically elevated emotional distress (ie, depression and anxiety symptoms) is common for both dyad members after ADRD diagnosis, which can become chronic and negatively impact relationship functioning, health, quality of life, and collaborative management of progressive symptoms.

**Objective:**

This study is part of a larger study that aims to develop, adapt, and establish the feasibility of Resilient Together for Alzheimer Disease and Related Dementias (RT-ADRD), a novel dyadic skills-based intervention aimed at preventing chronic emotional distress. This study aims to gather comprehensive information to develop the first iteration of RT-ADRD and inform a subsequent open pilot. Here, we describe the proposed study design and procedures.

**Methods:**

All procedures will be conducted virtually (via phone and Zoom) to minimize participant burden and gather information regarding feasibility and best practices surrounding virtual procedures for older adults. We will recruit dyads (up to n=20) from Mount Sinai Hospital (MSH) clinics within 1 month of ADRD diagnosis. Dyads will be self-referred or referred by their treating neurologists and complete screening to assess emotional distress and capacity to consent to participate in the study. Consenting dyads will then participate in a 60-minute qualitative interview using an interview guide designed to assess common challenges, unmet needs, and support preferences and to gather feedback on the proposed RT-ADRD intervention content and design. Each dyad member will then have the opportunity to participate in an optional individual interview to gather additional feedback. Finally, each dyad member will complete a brief quantitative survey remotely (by phone, tablet, or computer) via a secure platform to assess feasibility of assessment and gather preliminary data to explore associations between proposed mechanisms of change and secondary outcomes. We will conduct preliminary explorations of feasibility markers, including recruitment, screening, live video interviews, quantitative data collection, and mixed methods analyses.

**Results:**

This study has been approved by the MSH Institutional Review Board. We anticipate that the study will be completed by late 2023.

**Conclusions:**

We will use results from this study to develop the first live video telehealth dyadic resiliency intervention focused on the prevention of chronic emotional distress in couples shortly after ADRD diagnoses. Our study will allow us to gather comprehensive information from dyads on important factors to address in an early prevention-focused intervention and to explore feasibility of study procedures to inform future open pilot and pilot feasibility randomized control trial investigations of RT-ADRD.

**International Registered Report Identifier (IRRID):**

PRR1-10.2196/45532

## Introduction

### Background

Alzheimer disease and related dementias (ADRDs) are already experienced by 55 million individuals worldwide, and this number is projected to increase to roughly 75 million by 2030 [1]. ADRDs produce early symptoms (eg, increased forgetfulness, communication challenges, loss of recognition of places, time, and routines) that progress over time and undermine the independence, health, well-being, and relationships of persons living with ADRDs and their family care-partners [2-4]. Over 11 million family members are primarily responsible for supporting persons living with dementia for activities of daily living, and many are romantic or spousal care-partners [1,5,6]. Spousal care-partners are often considered “the second patient” [1] due to their shared experience of the life disruptions and stress experienced as a result of ADRDs and their instrumental role in assisting in medical care, daily activities, and transitions in functional independence [7-9]. Among care-partners, spouses and romantic partners are recognized as an important group to target for early support given their role in at-home caregiving activities, experience of relational disruptions, and greater likelihood of experiencing mental and physical health consequences of caregiving [6].

From symptom onset to receiving a formal or suspected diagnosis, both persons living with dementia and their spousal care-partners experience substantial changes in roles, plans, and expectations [8,10,11]. While some adjust well to these disruptions, many individuals (23%-52%) and spousal care-partners (35%-50%) experience “clinically elevated emotional distress” (ie, depression and anxiety) in the weeks and months leading up to and after diagnosis [12-17]. Dyads’ distress amplifies amid the progressive symptoms and lack of sufficient treatments for ADRDs and the impact on their daily life and relationships [18-20]. Dyads’ distress is also interdependent (ie, mutually experienced and bidirectional) and interferes with their ability to effectively communicate and problem-solve challenges [12,18,21,22]. Without adequate support or treatment, emotional distress is likely to become chronic and negatively impact dyads’ relationship functioning, mental and physical health, and quality of life [12,22,23]. At present, individuals living with ADRDs and their care-partners have little support available after diagnosis to manage stressors, cope with distress, communicate effectively, and collaboratively plan for the future [22,24]. There are no established treatments to date that substantively alter the course of ADRDs and no available psychosocial interventions to promote positive dyadic adjustment to challenges experienced early after diagnosis [11,25].

Both individuals living with ADRDs and their spousal care-partners describe having insufficient support and a lack of resources to assist them in managing early symptoms, expressing personal needs, and learning to address shared challenges together [26,27]. Dyads each express an interest in participating in psychosocial interventions together in order to learn skills to cope with the diagnosis and symptoms and prepare for future challenges [27-31]. Dyadic interventions exist for early-late stage ADRDs that demonstrate promise in reducing dyads’ emotional distress and neuropsychiatric symptoms in the person living with ADRD [32]. However, no available interventions target the period early postdiagnosis, thereby missing the window of opportunity to meaningfully include persons with ADRDs in collaborative treatment and planning before symptoms progress [11,33,34].

Dyadic interventions delivered early after ADRD diagnoses may be an optimal avenue for promoting adjustment to the challenges experienced by persons living with dementia and their spousal care-partners while they are still able to engage collaboratively in treatment and care planning. Such approaches are capable of simultaneously providing both partners with individual and interpersonal skills training to (1) effectively cope with adversity, thereby reducing emotional distress, and (2) preserve the quality of life by maintaining identity and normalcy, preserving social connections, and communicating to better navigate shared challenges and long-term care plans [35]. Dyadic interventions are more effective and economical than those focused on patients or care-partners alone [36] and are feasible and demonstrate some positive effects on the dyadic relationship in the context of early-stage ADRD [26]. Recent advances in telehealth approaches also allow these interventions to be delivered via live video telehealth (eg, Zoom), which can increase access and equity in care and allow for the personalization of skills-based interventions. Dyads coping with ADRD report similar preferences for telehealth and in-person visits, with fewer barriers using telehealth technology [37-39].

### This Study

Early dyadic interventions have the potential to promote positive adjustment to ADRD by improving dyads’ individual and interpersonal resiliency (ie, the ability to adjust effectively to significant adversity) [40] and provide skills and support to actively contribute to care-planning, preserve their autonomy, and express needs and preferences [41,42]. Many dyadic interventions include resiliency skills linked to positive adjustment after stress and trauma, including (1) mindfulness—staying present and deferring judgment in the face of adversity [43], (2) coping—building and applying cognitive, behavioral and emotional strategies to navigate challenges [44], (3) dyadic coping—open dialogue about stressors, preferences, and needs; approaching challenges as a unit [45,46], (4) social support—engaging social resources to meet needs [47], and (5) general self-efficacy (GSE)—perceived resourcefulness to adapt to adversity [48].

**Figure 1:**
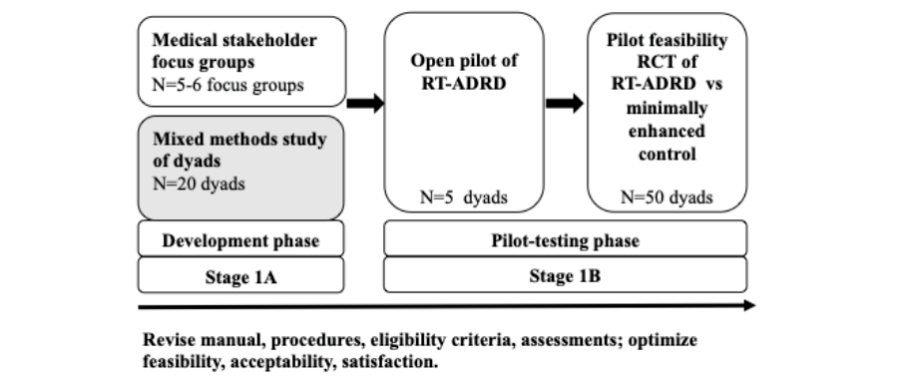
Proposed iterative development of RT-ADRD. RCT: randomized controlled trial; RT-ADRD: resilient together for Alzheimer disease and related dementias.

Members of our team developed Recovering Together, a brief dyadic resiliency intervention that prevents chronic emotional distress in patients and informal care-partners shortly after intensive care unit admission for acute neurological conditions (eg, traumatic brain injury, stroke) [49,50]. This study is part of a larger 5-year study that aims to use the prior research, methodology, program content, and procedures of the Recovering Together program as a basis for developing the novel Resilient Together for Alzheimer Disease and Related Dementias (RT-ADRD). RT-ADRD will be developed sequentially using the National Institutes of Health stage model and prior research to guide intervention development [9,51]. The purpose of this paper is to describe the protocol for a mixed methods study involving patient and spousal dyads early after ADRD diagnosis that will inform the development of the first version of RT-ADRD (Figure 1). Through this study, we hope to gather comprehensive information on dyads’ challenges, needs, and intervention preferences to develop a program that is feasible, acceptable, and capable of meaningfully improving target outcomes for persons living with dementia and their spousal care-partners early after ADRD diagnoses.

## Methods

### Ethics Approval

This study was approved by the Mount Sinai Hospital (MSH) Independent Review Board (22-01623).

### Study Design

**Figure 2:**
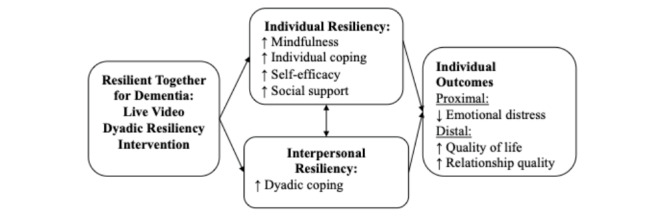
Study conceptual model.

We are currently conducting a mixed methods investigation involving persons living with ADRD and their romantic or spousal care-partners (target N=up to 20 dyads; recruitment scheduled to begin April 2023). This study is designed to (1) gather impressions of dyads’ challenges and needs early after ADRD diagnosis, and (2) obtain feedback on proposed RT-ADRD program procedures (eg, screening, recruitment, consent, intervention delivery, survey assessment) and intervention content. Quantitative assessment measures will be used to explore the feasibility of assessment procedures and gather data to explore preliminary associations among hypothesized intervention mechanisms and target outcomes (see Figure 2 and Table 1).

**Table 1:** Study measures.

Construct		Measure or description and type
**Covariates**		
	Demographics	Medical chart review and participant self-report: gender, age, race, ethnicity, marital status, education level, employment (status, occupation, and income), mental health history
	Clinical characteristics	Medical chart review and participant self-report: type of ADRD^a^ diagnosis, age at diagnosis, date of diagnosis, symptoms
**Primary outcomes**		
	Emotional distress	Hospital Anxiety and Depression Scale (14 items) (depression and anxiety subscales)–emotional distress Geriatric Depression Scale–Short Form (15 items)–depression Perceived Stress Scale (4 items)–stress
	Quality of life	World Health Organization Quality of Life–Short Form (26 items)–quality of life
	Relationship satisfaction	Couple Satisfaction Index (16 items)–relationship satisfaction and communication
**Primary intervention targets**		
	Mindfulness	Cognitive and Affective Mindfulness Scale–Revised (12 items)–mindfulness skills
	Individual coping	Brief Coping Orientation to Problems Experienced (28 items)–adaptive and maladaptive coping strategies
	Dyadic coping and communication	Dyadic Coping Inventory–common dyadic coping subscale (5 items) and negative dyadic coping (4 items) subscale–dyadic stress management Dyadic Relationship Scale (10 items for patients, 11 items for caregivers)–relationship strain
	Social support	Social Support Questionnaire-Short Form–Revised (12 items)–social support availability and satisfaction
	Self-efficacy	General Self-Efficacy Scale (10 items)–self-efficacy
	Caregiver readiness	Preparedness for Caregiving Scale (8 items; caregiver only)–preparedness for caregiving

^a^ADRD: Alzheimer disease and related dementias.

### Inclusion and Exclusion Criteria

We will recruit dyads with English fluency and literacy who are willing to participate and comply with the requirements of the study (including surveys and an interview). Eligible persons living with ADRD will be individuals aged 65 years or older who have a chart-documented ADRD diagnosis within the past month, ADRD symptom onset after the age of 65 years, and cognitive assessment scores and symptoms consistent with early-stage dementia (as determined by provider impressions and test scores such as the Clinical Dementia Rating Scale [52] scores of 0.5 or 1.0 or Montreal Cognitive Assessment [53] scores between 18-21). To be included in the study, they will also be required to (1) demonstrate cognitive awareness of their problems (as determined by the treating providers), and (2) capacity to understand the study and research protocol (confirmed during study screening using scores >12 on the University of California Brief Assessment of Capacity to Consent [UBACC] 10-item scale [54], described in detail below). In addition, at least 50% of dyads will exhibit clinically elevated emotional distress (as determined by self-report measures listed below) in order to compare dyads’ responses by level of distress and inform future selection criteria. Exclusion criteria for persons living with dementia are (1) a diagnosis of frontotemporal dementia-behavioral variant or other ADRD variant that would interfere with the ability to meaningfully participate, (2) inability to provide informed consent due to cognitive or behavioral impairments, and (3) being deemed inappropriate to participate by the referring providers. Additional exclusion criteria for dyads are comorbid terminal illness diagnosis in either partner. Both dyad members must be eligible and provide informed consent in order for either member of the dyad to participate.

### Recruitment and Screening

Dyads will be recruited via the MSH dementia care clinical infrastructure, including the departments of neurology, psychiatry, and geriatrics. The recruitment team will present the aims to medical staff in department clinics, explain the purpose and potential benefits of participation, and discuss ways to best facilitate referrals. As part of routine procedures, treating providers conduct assessments of each patient’s understanding (knowledge of facts) and appreciation (recognition of factors that apply to that person) of the presence and severity of cognitive impairment. Results of these assessments are included in patient’s medical records, and can be used as a metric for facilitating appropriate referrals. Treating providers will confirm diagnoses of ADRDs and clear patients for participation in the study before facilitating referrals. Potential participants will first hear about the study from their treating providers, who will introduce the study around the time of diagnostic disclosure and provide interested dyads with a study flyer. Providers will also obtain permission for the dyad to be contacted by the research team for recruitment purposes.

With dyads’ permission, providers will refer potentially eligible patients by sharing relevant dyad information with the study team, including (1) patient and spouse names and contact information, (2) medical record numbers, (3) diagnosis, and (4) date of diagnosis. Dyads can also contact the study team directly via a screening survey accessed through the study flyer on the Research Electronic Data Capture (REDCap) program. The research team will contact dyads via email to complete screening procedures using the REDCap survey and a screening call (Zoom video or telephone). The screening call will be used to provide dyads with more information about the study, answer questions, and assess eligibility. A member of the research team will speak with persons living with ADRD and spousal care-partners individually while administering initial screening measures to ensure privacy. Once determined eligible on these initial measures, dyads will have the option to complete the remaining screening and consent procedures individually or together according to preference. If either dyad member is deemed unable to meaningfully participate in the study based on screening procedures (eg, assessment of capacity to consent, described below) but is eligible based on the remaining criteria, the study staff will provide the dyad with a resource sheet containing local and national resources for ADRDs. If eligible, a member of the research team will document informed consent for dyads using an e-consent feature on REDCap.

### Assessment of Capacity to Consent

Individuals’ ability to understand the study and research protocol will be determined by a standardized teach-back method. Research staff will receive extensive training and supervision to administer the assessment, which will consist of the 10-item UBACC scale [54]. Scores >12 indicate ability to consent. After reviewing the consent form, individuals will be asked to repeat aspects of the consent back to the research staff. We will ask 4 questions about the expectations and risks of the study for participants, which have predetermined acceptable answers. Individuals who respond with 100% accuracy will be eligible to consent to the study, whereas individuals who do not achieve 100% accuracy will be prompted to reread the consent form and will be reasked the questions. If the individual does not achieve 100% accuracy a second time, they will not be eligible for the study.

### Qualitative Data Collection

After both dyad members consent to participate in the study, a member of the research team will schedule a 60-minute joint interview with a PhD-level clinical psychologist with expertise in conducting dyadic interviews. In advance of the interview, we will send dyads an interview guide with potential discussion topics and tips for using the secure live video software. A member of the research team will also be available to provide technology support and orientation to Zoom before and during the scheduled interview session. During the 60-minute interview, the clinical psychologist will follow a semistructured interview guide (see Multimedia Appendix 1). Another member of the study team will attend the interview to complete a rapid data analysis template developed by the research team. The rapid data analysis template will be used to inform formal coding and will capture observations within key interview domains, poignant participant quotes, researcher reflexivity, and important notes and observations (see Multimedia Appendix 2). During the interview, the clinical psychologist will work to generate information from dyad members on their convergent and divergent perspectives regarding their early experiences after ADRD diagnoses and unmet support needs and to gather feedback surrounding the proposed intervention content and procedures. Following the completion of the joint interview, dyad members will have the opportunity to participate in optional 15-minute individual interviews to share anything that they would prefer to discuss with the research team without their partner present.

### Quantitative Data Collection

#### Overview

Following the completion of the qualitative interviews, we will send each dyad member a quantitative survey to complete individually via the web-based REDCap platform which will take approximately 30-50 minutes to complete. Dyad members will have the option to complete the survey independently or over Zoom with assistance from a member of the research team. If completing the survey over Zoom, the research team member will use the “Share Screen” feature to gather responses to survey items and progress through the questionnaires. Questionnaires were selected to gather additional information surrounding the hypothesized intervention targets (ie, mechanisms of change), and outcomes, as well as selection criteria for individuals to target in the context of an early dyadic intervention. We selected questionnaires for the survey that each have strong psychometric properties and were feasible and acceptable in prior dyadic studies. Survey responses will be stored on the REDCap platform and accessed via password-protected laboratory computers. Paper data files (with coded subject identification) will be stored in a locked filing cabinet accessible only to the research team.

#### Demographics

We selected demographic variables to assess factors that may impact dyad experiences and adjustment to ADRD diagnoses. We will collect information on participants’ gender, age, race, ethnicity, marital status, education level (number of years in school), employment (status, occupation, and income), and mental health history.

#### Clinical Characteristics

We selected clinical characteristics to assess dyads’ experiences in the context of their ADRD symptoms and medical care. For the person living with dementia, we will collect information on their ADRD diagnosis and symptoms via electronic medical records, including clinical characteristics, type of diagnosis, age at diagnosis, and date of diagnosis.

#### Proposed Outcomes

We selected proposed outcomes based on prior research documenting the prevalence and correlates of emotional distress and low quality of life among dyads experiencing dementia and recent calls for interventions to promote positive adjustment early after diagnosis. We will use the 14-item Hospital Anxiety and Depression Scale (HADS) [55] total score to assess emotional distress. Scores range from 0-42 with higher scores indicating more emotional distress. We will also examine the 7-item depression and anxiety subscales of the HADS (HADS-D and HADS-A, respectively). Scores range from 0 to 21, with higher scores indicating more depression or anxiety. We will also use the 15-item Geriatric Depression Scale (GDS)–Short Form [56] to assess the past week depression symptoms. Scores range from 0-15, with higher scores indicating more depression. We will assess perceived stress over the past month using the perceived stress scale (PSS-4) [57]. Scores range from 0-16, with higher scores indicating greater perceived stress. To assess perceived quality of life, we will use the 26-item brief World Health Organization Quality of Life (WHOQOL-BRIEF) assessment [58]. The WHOQOL-BRIEF assesses quality of life across 4 subscales (physical health, psychological, social relationships, and environment). The first 2 items provide a global assessment of the quality of life, and subscale scores are calculated by summing items and transforming scores to a 0-100 point interval, with higher scores indicating greater perceived quality of life. To assess dyadic relationship satisfaction, we will use the 16-item Couple Satisfaction Index (CSI) [59]. Total scores range from 0-81, with higher scores indicating greater relationship satisfaction.

#### Treatment Targets

We selected treatment targets assessing individual and dyadic coping and relationship functioning based on our hypothesized mechanisms underlying changes in proposed outcomes (ie, emotional distress, quality of life). We will use the 12-item Cognitive and Affective Mindfulness Scale, Revised (CAMS-R) [60] to assess mindfulness. Total scores range from 12-48 with higher scores indicating greater engagement in mindfulness practices. We will use the 28-item Brief Coping Orientation to Problems Experienced (Brief COPE) [61] to measure ways of individually coping with stressful events. The Brief COPE includes 3 subscales that assess coping styles: (1) problem-focused (8 items), (2) emotional-focused (12 items), and (3) avoidant coping (8 items). Scores for each subscale are calculated with the sum of item scores divided by the number of subscale items, which higher scores indicating a greater degree of using the subscale coping style. To assess perceptions of dyadic coping, we will use the items from the Dyadic Coping Inventory [62,63] subscales: common dyadic coping and negative dyadic coping. The common dyadic coping subscale includes 5 items that assess dyads’ ability to cope with problems together and search for solutions. Scores range from 5-25, with higher scores indicating greater perceptions of the dyad’s use of common coping. We will also use the 4 items of the negative dyadic coping subscale of the Dyadic Coping Inventory to assess negative coping interactions. Scores range from 4-20, with higher scores indicating greater perceptions of the dyad’s use of negative dyadic coping. In addition, we will assess dyadic relationship strain in the context of caregiving using the dyadic strain subscale of the Dyadic Relationship Scale (DRS) [64], which has a 10-item patient version and an 11-item caregiver version. Subscale scores range from 1-10 and 1-11 for DRS-Patient and DRS-Caregiver, respectively, with higher scores indicating greater relationship strain. We will use the 12-item brief Social Support Questionnaire (SSQR) [65] to assess social support availability and satisfaction. The SSQR consists of 2 subscales: support network availability (SSQR-N) and overall satisfaction (SSQR-S). Each item assesses support availability and satisfaction in separate parts; participants will be asked to (1) indicate how many people they can count on for various types of support, and (2) indicate how satisfied they are with the support. The SSQR-N subscale composite is calculated by adding the total number of people for all items and dividing it by the total number of items for an average support number. For the SSQR-S subscale total scores are calculated by adding subscale items and range from 6-36, with higher scores indicating greater social support satisfaction. We will assess perceived self-efficacy with the 10-item GSE [66] scale. Total scores range from 10-40, with higher scores indicating greater perceived self-efficacy. Finally, for spousal care-partners we will use the 8-item Preparedness for Caregiving scale to evaluate readiness for caregiving in relation to their spouse. Total scores range from 0-32 with higher scores indicating greater preparedness for caregiving.

### Qualitative Analyses

Audio data from dyadic and individual interviews will be transcribed verbatim using a transcription service and then deidentified. We will upload and analyze deidentified transcripts using the Dedoose qualitative analysis software [67]. Our approach to data analysis will involve a hybrid deductive-inductive approach that will allow us to explore our key research aims surrounding experiences and treatment preferences of spousal dyads early after ADRD diagnosis. Specifically, our approach will be deductive, in the sense that our interview guide, rapid data analysis template, and codebook domains will be influenced by (1) prior research on early psychosocial challenges following ADRD diagnosis, (2) prior studies focused on dyadic intervention development and adaptation, and (3) theories of dyadic adjustment to chronic and progressive illness. For example, our interview guide will include questions surrounding dyads’ perceptions of available psychosocial supports, which we will explore further using a-priori defined rapid data analysis template and codebook domains. The remainder of our approach will be primarily inductive in nature, in the sense that our research team will document observations during rapid data analysis in a generative manner based on dyads’ discussion. We will refine our initial codebook using these observations and assess when thematic saturation is reached for key aims. Formal qualitative data analysis will then be guided by the framework method. Members of the research team involved in qualitative analysis will begin with line-by-line review of 1-2 dyadic interview transcripts and open code poignant observations, which we will organize into the a-priori deductive domains and revise our initial codebook through team discussion. Next, 2 members of the research team will code 25% (approximately 5/20) of the interview transcripts (n=5) in the Dedoose software package [67] during the initial codebook. Coders will resolve discrepancies through discussions in weekly meetings with the larger research team. We will ensure high agreement between coders before they code transcripts individually. We will then refine coded data into themes and subthemes within each domain through team discussion.

### Quantitative Analyses

We will use descriptive statistics to characterize the number and proportion of individuals approached, screened for eligibility, screened eligible, consented, and retained through interviews and surveys. For proposed outcome measures and treatment targets, we will calculate means and standard deviations of scores for each dyad member, and average scores of each dyad member to assess average responses across dyads. We will examine preliminary associations between treatment primary targets and emotional distress and quality of life outcomes using bivariate correlations. In addition, we will conduct a preliminary assessment of dyadic interdependence (similarity in patterns of responses) by calculating bivariate correlations of dyad members’ scores on survey composites.

### Comparison and Combined Interpretation of Qualitative and Quantitative Findings

We will use a convergent parallel mixed methods approach to evaluate and interpret qualitative and quantitative findings simultaneously [68,69]. This design involves concurrent, separate collection and analysis of qualitative and quantitative data followed by a detailed examination of convergence or divergence between qualitative and quantitative findings. Specifically, we will examine whether qualitative themes surrounding couples’ perception of psychosocial stressors correspond with perceptions on relevant quantitative measures (described as triangulation of findings; eg, with survey constructs such as emotional distress, quality of life, individual and dyadic coping). We will also examine whether correlations between quantitative measures are explained by qualitative descriptions of underlying processes. If observed, divergent findings will be further explored by examination of dyads’ demographic and clinical characteristics as well as through the integration of knowledge from prior research, consistent with guidelines for mixed methods research [70].

## Results

This study is funded by the National Institute on Aging grant 1K23AG075188 to SMB, who recently moved to MSH. This study was approved by the institutional review board of MSH. Recruitment started in April 2023. Data collection is anticipated to be completed by September 2023, and data analysis is anticipated to be completed by November 2023.

## Discussion

Receiving a diagnosis of ADRDs can have a devastating impact on individuals and their care-partners given the lack of available ameliorative interventions or cures and insufficient support early postdiagnosis [18,34]. Prior research suggests that (1) dyads of individuals and their care-partners are interested in participating in early psychosocial interventions together, and (2) that it is feasible to adapt existing dyadic interventions to effectively and efficiently address early challenges to dyads following an ADRD diagnosis [26,32,51]. Comprehensive feedback from dyads can facilitate the development of such programs, and be used to promote alignment between patient, caregiver, and health care system perspectives on ways of optimizing ADRD clinical care.

This paper describes a mixed methods study design that is part of the development phase of a larger study that aims to develop a novel postdiagnosis dyadic resiliency intervention for couples facing ADRDs. We describe the study aims and details of the procedures for recruitment, screening, data collection, and data analysis. This description is valuable for future studies focused on the adaptation and tailoring of dyadic interventions for ADRDs as well as other chronic and progressive conditions. Results of this trial will critically support the development of the first version of the proposed RT-ADRD intervention content and procedures. In addition, findings can be used in conjunction with our prior study of ADRD medical stakeholders to elucidate ways of integrating the RT-ADRD intervention and other supports within existing clinical infrastructures, and to navigate barriers and facilitators to dyads’ recruitment and participation in the RT-ADRD intervention. Our approach is consistent with National Institutes of Health stage model recommendations [9] to leverage findings to guide the development and refinement of program content and procedures prior to further testing.

This mixed methods study aims to gather comprehensive information from persons living with dementia and their spousal care-partners surrounding their experiences, unmet support needs, and feedback on proposed intervention content and procedures for a postdiagnosis dyadic intervention. The overarching goal is to inform the design, procedures, and content of the novel RT-ADRD program prior to subsequent pilot testing, clinical trials, and implementation efforts. Findings from this study also have the potential to increase available information surrounding dyads’ experiences following ADRD diagnosis and perceptions of helpful supports, which has implications for ADRD clinical care and can extend to other chronic and progressive neurological conditions.

## Appendix

Multimedia Appendix 1 Rapid data analysis template.

Multimedia Appendix 2 Interview guide.

Multimedia Appendix 3 Peer-review report by the Career Development for Clinicians/Health Professionals Study Section, National Institute on Aging (National Institutes of Health, USA).

## Abbreviations

ADRD: Alzheimer disease and related dementias
Brief COPE: Brief Coping Orientation to Problems Experienced
CAMS-R: Cognitive and Affective Mindfulness Scale, Revised
CSI: Couple Satisfaction Index
DRS: Dyadic Relationship Scale
EMR: electronic medical record
GDS: Geriatric Depression Scale
GSE: general self-efficacy
HADS: Hospital Anxiety and Depression Scale
MSH: Mount Sinai Hospital
PSS-4: Perceived Stress Scale
REDCap: Research Electronic Data Capture
RT-ADRD: Resilient Together for Alzheimer Disease and Related Dementias
SSQR: Social Support Questionnaire
UBACC: University of California Brief Assessment of Capacity to Consent
WHOQOL: World Health Organization Quality of Life

## Data Availability

Data collection and analysis instruments are available in Multimedia Appendices 1 and 2. The data sets generated or analyzed during this study are available from the corresponding author upon reasonable request.
