# Supplementary material for: A Live Video Dyadic Resiliency Intervention to Prevent Chronic Emotional Distress Early After Dementia Diagnoses: Protocol for a Dyadic Mixed Methods Study
Source: JMIR Res Protoc. 2023 Sep 20;12:e45532. doi: 10.2196/45532 (PMC10551792; doi:10.2196/45532)
Supplement: Multimedia Appendix 1 [file resprot_v12i1e45532_app1.docx]

| **Date:**  **Prepared by:**  **Interviewer:** |
| --- |
| **Dyad information**  **Diagnosis Date and Symptoms:**  **Dyad relationship characteristics (Examples: where they live, how long they’ve been together, prior or current occupation, kids):**  **Other relevant characteristics** (who the person provides care to): |
| **Domain 1: Early Symptoms and Challenges** |
| **Domain 2: Changes in Relationship After Diagnosis** |
| **General Impressions** |
| **Program Content:** |
| **Additional Comments:** |
| **Program Barriers and Facilitators:** |
| **Important observations and reflections**  **Broad themes and topics of interest in the interview** (eg, things not covered in rapid data analysis domains)**:**  **Important quotations** |
| **Interviewee analytic notes + reflexivity** (eg, any aspects of researchers’ identity, beliefs, social positioning and how they might influence the interview content and observations—consider how your field notes are your own “interpretations” of the interactions that took place)**:** |
| **Behind-the-scenes information (before or after recording; nonverbal information; etc):** |
