# Supplementary material for: A Live Video Dyadic Resiliency Intervention to Prevent Chronic Emotional Distress Early After Dementia Diagnoses: Protocol for a Dyadic Mixed Methods Study
Source: JMIR Res Protoc. 2023 Sep 20;12:e45532. doi: 10.2196/45532 (PMC10551792; doi:10.2196/45532)
Supplement: Multimedia Appendix 2 [file resprot_v12i1e45532_app2.docx]

**Resilient Together Interview Topics**

Our team is interested in developing programs that support patients and their families early after diagnosis of Alzheimer or other forms of dementia (abbreviated as AD throughout this document). The National Institute of Aging is paying for this study.

We have talked to many patients and their loved ones thus far and learned about the challenges associated with dementia diagnoses. Specifically, we learned that the path to receiving a diagnosis is stressful for many people. Patients and their spouses describe how it can be difficult to share their thoughts and feelings with one another, and that they would like more resources to come to terms with the diagnosis and best prepare for the future.

Using this information, we have an idea of what the program might look like and some strategies that might be helpful. We hope to get your thoughts on the specific features of the program so that we can make it as useful as possible.

Below is a list of some of the topics we will discuss during our conversation.

**Early Symptoms and Diagnosis**

First symptoms you each noticed and how you communicated about them

How doctors delivered the diagnosis

Immediate reactions to diagnosis

Greatest support needs after diagnosis

Resources provided to you after diagnosis

**Changes in relationship with partner after diagnosis**

Changes have you observed in your relationship

Challenges you face with respect to communicating

Different communication styles

Different needs

**Program Recommendations**

We are working toward developing a program to support couples early after dementia diagnoses. This would be delivered in addition to other services available to couples.

This program would be to help couples:

1) communicate about their individual emotions, needs, and challenges

2) learn how to access support

3) understand ways of discussing the diagnosis with other family/friends

4) learn ways of discussing financial, legal, and healthcare issues, and

5) prepare for the future.

**General Impressions**

What are your first impressions of a program like this?

Would you be willing to participate in a program like this? Why or why not?

What would be the main reasons you would want to participate?

What else would you want this program to address?

If you were to have access to this type of program, what would you want to learn?

**Program Content**

We have some ideas of session topics in mind. We would like to know your thoughts on each of the potential sessions we present to see if either of you would find them useful.

We will ask you to describe your thoughts and reactions to a list of potential topics and skills that we would like to include in the program.

We also plan to teach several skills. “Skills” are things that you can do to get through a difficult situation or cope with challenging emotions.

**For each topic and skill, we will ask for your:**

1) familiarity with the topic/skill, and

2) opinions of how the topic/skill could help address your “**AD-related challenges**,” which we define as stressors or changes that you have noticed since AD symptoms began.

We will ask you both about your preferences and encourage you to share your impressions, even if you disagree.

1. **Adjusting to Life after Diagnosis:** We think that all couples would benefit from a review of educational information on AD (common symptoms, challenges, personal reactions) and relevant resources (such as information from National Institute on Aging).

What do you think about the topic? What would you want to learn about during this session?

1. **Staying in the Present Moment:** Early after diagnoses many people feel overwhelmed by worries about the future and new responsibilities. We would like to include skills to help you stay in the present moment so that you can better manage daily tasks.

What do you think about the topic of this session? What sorts of AD-related challenges do you think “staying in the present moment” would be helpful for?

1. **Mindfulness**: Mindfulness means paying attention, on purpose, to the present moment, without judgment. Regular practice of mindfulness can help us stay more present and be more aware of and better manage emotions and difficult thoughts.

What do you think about the topic of this session?

What sorts of AD-related challenges, if any, could you use this skill to address?

1. **Deep breathing** is another skill that can help you manage physical reactions associated with stress, anxiety, or fear. For example, taking slow breaths can help reduce heart rate and relax the body.

What do you think about the topic of this session?

How do you think deep breathing could help manage challenges you have experienced with AD?

1. **Coping with uncertainty**: One of the most challenging things about AD is the uncertainty of symptom progression, treatment options, and ways of planning for the future. We want to have a discussion around uncertainty, how it brings forward conflicting feelings and emotions as we try to think through scenarios of what might be, and how they are experienced by patients and spouses.

What do you think about the topic of this session?

What have you felt most uncertain about pertaining to AD and AD-related challenges?

1. “**Dialectical Thinking” or Acknowledging Multiple Truths:** This skill acknowledges that more than one thought, emotion, or thing can be true at the same time. For example, you may say “I feel angry about my diagnosis AND am grateful for the care I am receiving.”

What do you think about the topic of this session?

How do you feel this skill may be helpful in managing AD-related challenges, if at all?

1. **Navigating Acceptance versus Change-Based Coping:** This skill would help you determine what challenges can and cannot be changed. It would also help you learn to problem-solve what can be changed, such as ongoing responsibilities or daily hassles, and accept what cannot be changed, like the AD diagnosis.

What do you think about the topic of this session?

What challenges have you faced with understanding acceptance versus change-based coping since the onset of AD symptoms?

1. **Navigating Personal Relationships.** Couples have described how it can be difficult to discuss individual and relationship changes and openly share their individual thoughts and emotions. We would focus on how to best communicate and support each other.

What do you think about the topic of this session?

In what ways, if any, have you seen your personal relationships change since receiving the diagnosis?

1. **Communication about Individual Needs.** This skill enhances interpersonal communication by encouraging partners to be clear, intentional, empathetic, and positive when sharing needs and listening to others.

What do you think about the topic of this session?

In what ways do you think that this skill could help manage AD-related challenges?

1. **Navigating Communication Styles:** Knowing how each partner shares information about AD-related challenges can help to build on strengths and plan for conversations that may be more difficult to have.

What do you think about the topic of this session?

How have you noticed your communication as a couple changing since receiving the AD diagnosis, if at all?

1. **Identity and Role Changes:** An AD diagnosis can impact perceptions of identity and lead to changes in familiar roles and relationships. Changes in our relationships can impact day-to-day experiences together, so we would identify these changes and work on ways to help couples stay connected.

What do you think about the topic of this session?

How have you seen a shift in your identity and roles, if at all?

1. **Identifying Changes in How you View Yourself**, both alone and in relationships with others. We would use previously learned skills (such as **dialectics/ multiple truths**) to discuss ways of adapting to new roles (such as being a caregiver or care-recipient), while also maintaining roles and personal identities when possible (such as being a partner or friend). Couples would be encouraged to support each other through this process.

What do you think about the topic of this session?

What changes have you found the most challenging to adapt to?

1. **Grieving Losses and Adjusting to a “New Normal”:** Many people experience feelings of grief or struggle with “losing” out on parts of their life or with what they thought life would be like moving forward. This session would focus on coping with these feelings individually and together.

What do you think about the topic of this session?

What would you find most beneficial to discuss during this session?

1. **Medical Care and Healthy Lifestyle Behaviors:** Many people have trouble discussing needs and preferences for medical appointments, understanding how to follow doctors’ suggestions, and practicing healthy self-care routines and behaviors. This topic would focus on ways of discussing needs and preferences and setting realistic expectations individually and together.

What do you think about the topic of this session?

What aspects of coordinating medical care, if any, have been the most challenging to manage so far?

1. **Setting Specific Goals:** One skill can involve strategies to keep goals simple and doable to help with everyday tasks and manage AD-related challenges.

What do you think about this skill?

What areas of your life would you be most interested in using this skill for?

1. **Identifying Values and Promoting a Positive Outlook:** Given the uncertainty about the future, many report that living a meaningful life is particularly important. This topic would involve thinking about current values and priorities and ways of making small changes to live a life that is meaningful. It would also address positive coping skills, such as using humor or cultivating optimism.

What do you think about the topic of this session?

How do you feel like your values have shifted since receiving the diagnosis, if at all?

What does “promoting a positive outlook” mean to you, and has this meaning changed since receiving the diagnosis?

1. **Fear Related to the Future:** Fear, particularly concerning symptom progression, can create more stress and prevent couples from engaging in the present moments their life. This topic would build off other skills of staying present (mindfulness), accepting what one cannot change and changing what is possible (dialectical thinking/multiple truths), and address ways of managing fears effectively.

What do you think about the topic of this session?

What fears, if any, would you want this session to address?

1. **Future Challenges:** This topic would discuss anticipating future challenges and then planning ways to cope ahead that would be helpful. We have found that balancing present-moment awareness with future planning can put people at greater ease when coping with stress.

What do you think about the topic of this session?

What sorts of challenges, if any, would you want to discuss during this session?

1. **Making Meaning:** One topic would discuss making meaning out of the experience of receiving an AD diagnosis, such as focusing on what this experience means for each person and the relationship. Participants would be encouraged to think about changes in the way they are treated, how their everyday life may have changed, and what impact it has had on their perspective or spiritual beliefs.

What do you think about the topic of this session?

What sorts of challenges, if any, could this skill be helpful for?

1. **Reflection:** A specific skill of “making meaning” would include **reflecting** on the experience in depth. This skill would build off other skills of observing and describing what has changed and stayed the same (mindfulness and dialectical thinking) and discuss the benefits of reflection to make the most out of experiences.

What do you think about this skill?

What areas of your life have you, or have you not, been able to reflect on since receiving the diagnosis?

How has this reflection (or lack thereof) made you feel?

1. **“Bucket List”:** Couples would also be encouraged to make a **“bucket list”** of how they spend their time which could include fun or meaningful activities, relationships, or individual goals.

What do you think about the topic of this session? What sorts of challenges, if any, could you use this skill for, and how do you think this would help you manage the diagnosis?

**Additional Comments**

Are there **additional** topics or skills would be helpful to learn and practice together?

What specific topics or skills would be most helpful for you both to cover together?

Are there sessions that we discussed or additional topics you would prefer to talk about alone?

Would you be interested in any potential additional sessions involving other members of your family?

**Program Barriers and Facilitators**

What might prevent you from participating in a program like this?

What would help you to be able to attend?

What do you think about attending together versus separately?
